# Global Change Factors Influence Plant-*Epichloë* Associations

**DOI:** 10.3390/jof9040446

**Published:** 2023-04-06

**Authors:** Daniel A. Bastías, Andrea C. Ueno, Pedro E. Gundel

**Affiliations:** 1 AgResearch Limited, Grasslands Research Centre, Palmerston North 4442, New Zealand; 2 Centro de Ecología Integrativa, Instituto de Ciencias Biológicas, Universidad de Talca, Talca 3480094, Chile; 3 Instituto de Investigación Interdisciplinaria (I3), Universidad de Talca, Campus Talca, Talca 3480094, Chile; 4 Facultad de Agronomía, IFEVA, CONICET, Universidad de Buenos Aires, Buenos Aires C1417DSE, Argentina

**Keywords:** climate change, endophyte, stress, symbiosis, phytohormone, ROS, antioxidant, transgenerational effect

## Abstract

There is an increasing interest in determining the influence of global change on plant–microorganism interactions. We review the results of experiments that evaluated the effects of the global change factors carbon dioxide, ozone, temperature, drought, flooding, and salinity on plant symbioses with beneficial *Epichloë* endophytes. The factors affected the performance of both plants and endophytes as well as the frequency of plants symbiotic with the fungus. Elevated carbon dioxide levels and low temperatures differentially influenced the growth of plants and endophytes, which could compromise the symbioses. Furthermore, we summarise the plant stage in which the effects of the factors were quantified (vegetative, reproductive, or progeny). The factors ozone and drought were studied at all plant stages, but flooding and carbon dioxide were studied in just a few of them. While only studied in response to ozone and drought, evidence showed that the effects of these factors on symbiotic plants persisted trans-generationally. We also identified the putative mechanisms that would explain the effects of the factors on plant–endophyte associations. These mechanisms included the increased contents of reactive oxygen species and defence-related phytohormones, reduced photosynthesis, and altered levels of plant primary metabolites. Finally, we describe the counteracting mechanisms by which endophytes would mitigate the detrimental effects of the factors on plants. In presence of the factors, endophytes increased the contents of antioxidants, reduced the levels of defence-related phytohormones, and enhanced the plant uptake of nutrients and photosynthesis levels. Knowledge gaps regarding the effects of global change on plant–endophyte associations were identified and discussed.

## 1. Introduction

Global change is dramatically altering natural ecosystems and biodiversity. The global mean surface temperature is expected to increase by about 1.5 °C due to the elevated emissions of greenhouse gases and pollutants such as CO_2_ and ozone [1]. Climate is changing at local and regional scales, increasing the frequency and intensity of cold, heat, drought, and flooding events [1,2]. Salt contents in soil are also increasing as consequence of climate change and inadequate agricultural practices [3]. Evidence shows that the environmental factors associated with global change influence different aspects of the biology of plants including growth and reproduction [4]. Furthermore, the global change factors are challenging the production of major world-wide crops such as wheat, rice, maize, and soybean [5]. In natural and managed ecosystems, plants are normally associated with beneficial microorganisms that promote growth and plant fitness [6,7]. Given their critical role in plant fitness, there is an increasing interest to understand the effects of global change factors on the interaction of plants with beneficial microorganisms [8,9]. It is particularly interesting to determine if the global change factors alter the benefits conferred by microorganisms to their hosts and the mechanisms that underlie these alterations [7,10].

Plant–*Epichloë* associations are interesting symbioses to investigate the effects of global change on plants that interact with beneficial microorganisms. *Epichloë* fungi form endophytic associations with Pooideae grasses and inhabit intercellular spaces of green plant tissues [11]. Most of these endophytes are maternally inherited by establishing mycelia in mature seeds [12]. In these symbioses, the fitness of plants and endophytes are strongly aligned since host plant reproduction and seed stage provide the opportunity for symbionts to multiply and disperse [13]. Plants and vertically transmitted endophytes form mutualistic associations. The success of these symbioses (measured as frequency of symbiotic plants in populations) depends on both the net benefit conferred by endophytes on plants and the efficiency of vertical transmission [14,15]. *Epichloë* endophytes confer multiple benefits to their plant hosts, and the most documented is the antiherbivore protection given by endophyte-derived alkaloids [16]. *Epichloë* endophytes also alter the levels of phytohormones and induce the production of plant secondary metabolites that enhance the host tolerance against abiotic and biotic stress factors [17,18]. Additionally, the endophytes increase the contents of antioxidants in plants that help to mitigate the oxidative damage triggered by environmental stress factors [19]. Despite all these benefits, plant–*Epichloë* interactions can transiently turn into negative associations by either the action of certain stress-triggered plant responses or the limitation of plant resources (i.e., endophyte-symbiotic plants displaying lower fitness than their endophyte-free counterparts) [15,20]. As an expression of the context-dependent symbiosis outcome, global change factors are likely to affect the persistence, distribution, and abundance of plant–endophyte symbiosis in the near future.

The aim of this review is to describe some of the documented effects that global change factors exert on plant–*Epichloë* symbioses. The factors considered in the present work are carbon dioxide (CO_2_), ozone, heat, cold, drought, flooding, and salinity. Most, but not all, of the listed factors can generate stress and growth reductions in plants. For instance, within certain range, the environmental temperature can stimulate the growth of plants [21]. The factors were selected due to their recognised effects on plant fitness and the available information in the plant–endophyte literature [22,23]. We summarised published results showing the effects of the selected global change factors on plant–*Epichloë* associations, and identified the putative mechanisms that would explain the effects of these factors on the associations. Furthermore, we described the counteracting mechanisms by which endophytes would mitigate the detrimental effects of the global change factors on plants. For vertically transmitted endophytes, these mechanisms would be critical for their persistence in individual plants and plant populations. Our study contributes to understanding the effects of global change factors on plants that interact with endophytes, the specific mechanisms that explain these effects, and the endophyte-conferred mechanisms that counteract and alleviate the negative effects.

## 2. Effects of Global Change Factors on Plant–*Epichloë* Associations

**Figure 1:**
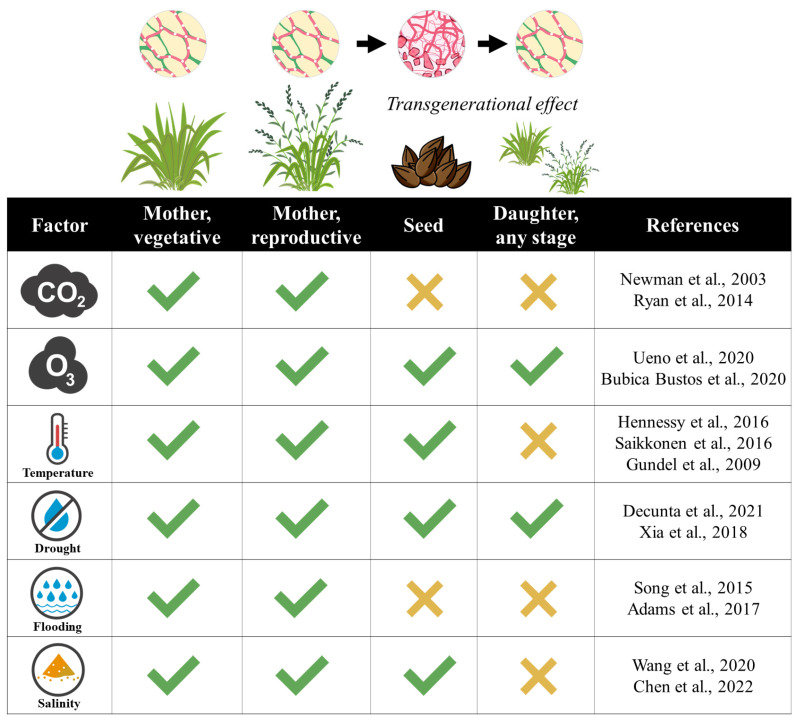
Summary of the presence/absence of experimental results evaluating the effects of global change factors on distinct stages of the lifecycle of plants associated with fungal endophytes. The top diagram shows plant and endophyte lifecycles. The plant lifecycle is divided into mothers (stages vegetative or reproductive), seeds, and daughters (at any stage). The endophyte lifecycle shows the presence of the fungus within tissues of mother, seed, and daughter plants and the fungal transmission from mothers to seeds and seeds to daughters (with horizontal black arrows). The ‘transgenerational effect’ refers to those effects exerted by the factors on mothers that persist in the progeny (seeds and/or daughters). The ✓ indicates the existence of studies that evaluated the effects of a given factor on the performance of plant hosts or endophytes in a particular plant lifecycle stage, whereas the ✕ indicates a lack of studies. Plant performance refers to growth, reproduction, or survival, and endophyte performance to growth, alkaloid production, survival, transmission, or frequency in plant populations. The global change factors were not necessarily applied at the same plant stage that the plant performance was measured (e.g., factor applied at seedling stage, but performance measured at reproductive stage). The column ‘References’ refers to articles that contain experimental results associated with the effects of the factors carbon dioxide (CO_2_), ozone (O_3_), cold and heat/warm temperatures, drought, flooding, and salinity on plant–endophyte symbioses [18,25,29,32,37,42,51,53,64,68,70,74,79].

The environmental factors associated with global change affect distinct processes and functions in both plants and endophytes across the plant life cycle. Since fungal hyphae grow vegetatively in newly formed host seeds, the effects of global change factors on symbiotic plants can be trans-generationally transmitted (Figure 1).

Multiple studies have shown that atmospheres with elevated CO_2_ levels influence plant–*Epichloë* associations by affecting the plant/endophyte growth and fungal production of alkaloids. High CO_2_ levels increased the biomass of *Festuca arundinacea* (Schreb.) (Syn. *Schedonorus arundinaceus*) and *Lolium perenne* plants associated with endophytes, but the greenhouse gas did not affect the production of reproductive tillers or seed in symbiotic plants [24,25,26]. Similar beneficial effects of CO_2_ on plant growth were documented in endophyte-symbiotic *Brachypodium sylvaticum* and *L. perenne* plants that grew in soils with high nutrient contents [27,28]. Experimental results showing positive effects of CO_2_ on endophytes have been also reported. Elevated CO_2_ levels increased the amount of endophyte mycelial biomass in *F. arundinacea* [29]. Furthermore, an increased frequency of endophyte-symbiotic plants was documented in *F. arundinacea* populations that were exposed for several years to high CO_2_ levels [30]. Only a few experimental results have shown negative effects of the greenhouse gas on plant–endophyte associations. Elevated CO_2_ levels reduced the fungal production of alkaloids and eased the endophyte-based plant growth promotion in the same plant species [29,30,31].

Tropospheric ozone influences plant–*Epichloë* associations by affecting host morphophysiological traits and the endophyte persistence within plants and populations. Irrespective of the plant symbiotic status, high ozone levels reduced the photochemical efficiency and leaf greenness in *L. multiflorum* plants, but the oxidative damage induced by the pollutant was generally lower in endophyte-symbiotic than non-symbiotic plants [32,33]. The symbiosis increased the survival of seedlings under elevated ozone levels, but the pollutant reduced the reproductive effort of symbiotic plants (the ratio between reproductive and shoot biomass) [32,33,34]. Reduced seed longevity was also documented in endophyte-symbiotic plants that grew in environments with high ozone levels [35,36]. While ozone did not affect the transmission efficiency of endophytes from plant to seed, the viability of the fungus declined at a faster rate in seed produced by plants exposed to the pollutant [32,35]. Ozone did not affect either the concentration of alkaloids nor the biomass of fungal mycelia within plant green tissues or seed [32,34]. Despite the lack of effect of the ozone on alkaloids, the level of resistance to herbivores in symbiotic plants was reduced by the pollutant, and this effect persisted in the next plant generation [34,37,38].

Cool and warm temperatures affect plant–*Epichloë* associations by altering the plant/endophyte growth and fungal production of alkaloids. Cool temperatures reduced the growth of grasses associated with endophytes [39]. Low temperatures also reduced the endophyte mycelial biomass and alkaloid concentrations in *F. arundinacea*, *L. perenne*, and *L. multiflorum* [40,41,42,43]. Furthermore, low temperatures diminished the frequency of endophyte-symbiotic plants in *F. arundinacea* populations [39]. This stress also decreased the concentration of alkaloids within plants and compromised the endophyte-based resistance to insects [42,44]. In opposition to low temperatures, the fitness of endophyte-symbiotic plants was generally increased by treatments with warm temperatures. In *F. arundinacea*, the warm temperature stimulated biomass production more in endophyte-symbiotic than endophyte-free plants [45]. Moreover, enhanced concentrations of certain endophyte-derived alkaloids were documented in *F. arundinacea* and *L. perenne* plants grown in warm temperatures [45,46,47,48], but see [40]. In field experiments, concentrations of endophyte-derived alkaloids were positively correlated with the environmental temperature experienced by plants [49,50]. Furthermore, the endophyte-mediated promotion in the number of plant flowerheads was apparently influenced by the variation in the temperature in conjunction with other environmental variables in the field (e.g., soil nutrient contents, water availability) [51]. High temperatures usually exert negative effects on the endophyte presence in seeds. The endophyte viability in seed is usually reduced in environments that combine elevated temperature and moderated to high relative humidity [52]. For example, endophytes were not viable when seed were exposed for 100 days to 40 °C and 43% of relative humidity (while the seed were 100% viable) [53]. 

Multiple studies have evaluated the effects of drought on plant–*Epichloë* associations. The general pattern is that endophytes increase the survival and stimulate the growth of plants subjected to this stress [18,54,55]. For instance, the endophyte presence increased the tillering of *F. arundinacea* plants under drought [56]. Similarly, the endophyte also stimulated the growth (and photosynthesis rate) of *Achnatherum inebrians* plants that experienced water restriction [57]. In the case of *L. multiflorum*, symbiotic plants exhibited high water use efficiency and root conductivity under drought, but plant growth was not affected by the fungus [58]. In addition to the effects on plants, drought generally increased the concentration of endophyte-derived anti-herbivore alkaloids [56,59,60]. The endophyte presence also influenced the host seed production in certain genotypes of *L. perenne* in drought situations [61]. Few experiments have shown negative effects of endophytes on plants subjected to drought. For example, reduced water availability inhibited the germination of endophyte-symbiotic seeds more than non-symbiotic seeds [62]. These effects vary in their magnitude—but seemingly not direction—depending on the species/genotypes of both the plants and endophytes [18,63]. Furthermore, the magnitude of the benefits conferred by *Epichloë* endophytes to plants in drought situations also depends on maternal effects in the host plants [64].

Compared to drought, the effects of *Epichloë* on plants experiencing flooding stress have been less well documented. This may be due to the fact that early experimental results did not find that the endophyte presence provided advantages to plants that experienced flooding (see [65]). Another reason could be that most of the early research was performed on plant species/genotypes that are already somewhat flood-tolerant (i.e., *F. arundinacea* and *L. perenne*) [66]. However, more recent investigations have shown that distinct plant–endophyte combinations behave differently in the presence of flooding. For instance, the endophyte enhanced the growth and leaf water contents in certain genotypes of *F. arundinacea* plants that experienced the stress [67]. Furthermore, *Hordeum brevisubulatum* plants naturally associated with endophytes showed higher foliar biomass than their non-symbiotic counterparts grown in soils with excess water [68]. A similar result was documented in distinct ecotypes of *Festuca sinensis*, where endophyte-symbiotic plants accumulated more biomass under flooding conditions than endophyte-free plants [69]. Less common are experimental results showing negative effects of this stress on endophyte-symbiotic plants. Reduced foliar biomass and seed production was documented in endophyte-symbiotic *Poa leptocoma* plants in flooding conditions [70]. However, the incidence of endophyte-symbiotic plants in the population was high, suggesting that other endophyte-derived benefits outweighed this apparent cost [71]. 

*Epichloë* endophytes generally increased the biomass and seed production of plants grown in soils with high salinity contents [72,73,74,75,76]. Furthermore, the endophyte also enhanced the survival and germination of seeds that experienced high salinity [77,78]. High salinity also increased the concentration of endophyte alkaloids and mycelial biomass within plant tissues [59,79]. 

## 3. Mechanisms Underlying the Effects of Global Change Factors on Plant–*Epichloë* Associations

**Figure 2:**
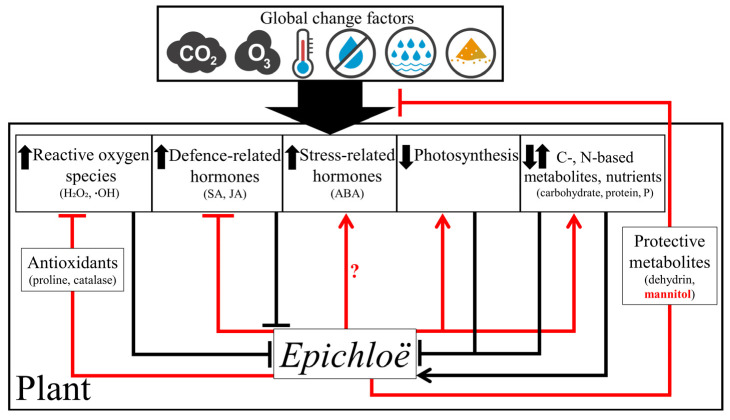
Mechanisms by which global change factors stimulate or inhibit endophyte fungi in plants and fungal mechanisms that counteract the negative effects. Certain factors increase the contents of carbon (C)-based primary metabolites that stimulate the endophyte growth in plants. Opposite to this, some factors enhance the amount of reactive oxygen species (ROS), defence-related hormones, and stress-related hormones, reduce photosynthesis levels, and diminish the contents of nitrogen (N)-based primary metabolites and nutrients that inhibit the growth of endophytes in plants and the fungal provision of benefits. Endophytes increase the contents of ROS-scavenging antioxidants, reduce the levels of defence-related hormones, induce photosynthesis, stimulate the plant acquisition of nutrients, and produce (or induce the plant production of) protective metabolites (e.g., dehydrin, mannitol) that potentially counteract/alleviate the detrimental effects of the factors. Arrows indicate positive regulation and truncated lines negative regulation. Black connectors show the effects of factors and plant processes on plants and endophytes. Red connectors denote endophyte effects on plant processes and plant-factor interactions. Endophyte-based metabolites are highlighted in red. The question mark indicates a putative endophyte regulation. The factors are carbon dioxide (CO_2_), ozone (O_3_), cold and heat/warm temperatures, drought, flooding, and salinity. Abbreviations: H_2_O_2_, hydrogen peroxide; ∙OH, hydroxyl radical; SA, salicylic acid; JA, jasmonic acid; ABA, abscisic acid; P, phosphorus.

The environmental factors associated with global change induce certain plant responses that may affect the presence of *Epichloë* endophytes and their derived benefits in plants (Figure 2).

Grasses hosting *Epichloë* endophytes are C3 species, and it is well documented that elevated CO_2_ levels stimulate the growth and photosynthesis of these species [80,81]. Higher concentrations of non-structural and soluble carbohydrates have been generally reported in C3 plants exposed to elevated CO_2_ levels [82]. This CO_2_-induced increase in carbohydrate contents may explain the documented growth stimulation observed in both plants and endophytes [29]. Concentrations of nitrogen compounds are usually reduced in plants grown in environments enriched with CO_2_ [81,82]. Since alkaloids are nitrogen-based compounds, low concentrations of endophyte-derived alkaloids reported in plants exposed to high CO_2_ levels could be explained by reduced nitrogen contents [29,83]. However, despite that the CO_2_ reduced the concentration of endophytic alkaloids, the fungus still conferred protection to the plant hosts against aphids [84]. A possible explanation for this outcome could be that the reduced alkaloid levels were still above the bioactivity thresholds [85]. Alternatively, CO_2_ could have reduced the quality and palatability of tissues or stimulated the accumulation of other compounds with anti-herbivory effects. In fact, plants grown in environments with elevated CO_2_ levels generally showed high concentrations of antiherbivore phenolic compounds [82]. 

The increased concentrations of reactive oxygen species (ROS) in plants triggered by ozone may explain, at least in part, the negative effects of this stress on plant–*Epichloë* associations [19,20]. ROS at high levels damage DNA, lipids, and proteins which can lead to cell death [86]. In addition to the oxidative damage on plants caused directly by ozone, altered ROS levels reduce the growth of endophytes within plant tissues [87]. Endophytes with mutations in enzymes that produce or regulate the production of ROS exhibited unrestricted growth within plant tissues but caused stunted and sometimes lethal phenotypes in their hosts [87,88]. ROS might also limit the distribution of endophyte mycelia within plant tissues due to their effects strengthening plant cell walls [89]. Ozone can also increase the levels of defence-related phytohormones such as salicylic acid and jasmonic acid [90]. These hormones negatively affect fungal endophytes since they induce the production of antimicrobial compounds by plants, deposition of callose in plant cell walls (that block the spread of the fungus), and programmed cell death [91,92,93,94].

Temperature stresses including both cold and heat increase the levels of ROS and cause oxidative damage in plant tissues [95]. The inhibition in endophyte growth documented in situations of temperature stress may be associated with increased ROS levels [42]. The defence-related phytohormone salicylic acid is also stimulated in situations of temperature stress [95]. This hormone affected the endophyte provision of benefits to plant hosts. The exogenous application of salicylic acid on plants reduced the concentration of fungal-derived alkaloids and promoted susceptibility of symbiotic plants against insect herbivores [96,97]. Another documented effect of low temperatures in plants is the reduced photosynthetic rate [98]. Variations in photosynthate levels, due to reduced photosynthesis, could also explain the documented changes in endophyte growth and alkaloid production within plants [43,45]. Alkaloid concentrations may also be affected by temperature-based changes in the kinetics of biosynthesis and degradation [99]. Furthermore, differences between plant and endophyte may explain the effects of the stress on the fungal growth and alkaloid production. For instance, *F. arundinacea* plants presented lower minimum cardinal temperatures than their associated endophytes (i.e., the lowest temperature at which an organism can grow) which suggests that at low temperatures, both fungal mycelia and alkaloids may be ‘diluted’ within plant tissues since only plants have maintained the growth [39]. 

The drought tolerance conferred by endophytes to plant hosts has been well-studied and excellent reviews have summarised and discussed the mechanisms [18,54,55]. Drought usually increases ROS levels, induces defence-related phytohormone responses, and reduces chlorophyl content in plants [100,101]. Similar to other stresses, *Epichloë* endophytes might be negatively affected by these plant responses. It is worth mentioning that the magnitude of the effects of the water deficit on plant–endophyte associations depends on the intensity and length of the event [63]. As indicated, results from short-term drought experiments showed that endophyte-symbiotic plants have a clear advantage in terms of plant performance over the non-symbiotic ones [18]. However, evidence from field surveys suggested that the plant capacity to host endophytes was impaired under extreme aridity [102,103]. 

Excess water in the soil causes hypoxia/anoxia in plant roots [104]. Although *Epichloë* endophytes are not found in roots, the negative consequences of flooding on host performance are likely to impair the symbiosis. Reduced chlorophyll contents, inhibited photosynthesis, and increased leaf senescence are some consequences of flooding on plants [105]. Furthermore, ethylene and ROS are generally accumulated within tissues when plants are subjected to flooding [106]. The reduction in photosynthesis rate may decrease the endophyte growth within plant tissues. Additionally, the fungal growth may be altered by the increased levels of ROS and phytohormones. Whereas no studies have evaluated the effects of flooding on the endophyte growth or its derived benefits, evidence from field studies suggest that the endophyte performance may be compromised under excess of water. For instance, a field survey found that *L. multiflorum* plants occurring in humid prairies recurrently subjected to flooding showed low endophyte transmission from plants to seed [107]. 

Salinity stress also increases the ROS levels in plants [108]. Similar to other stresses, altered ROS levels under salt stress may affect the growth of *Epichloë* endophytes [88,89]. The phytohormone jasmonic acid is increased in salt stress, and the induction of the defence responses associated with this hormone negatively affected the endophyte-derived benefits [109]. For instance, the exogenous application of methyl jasmonate (an activator of jasmonic acid defence responses) on symbiotic plants reduced the concentration of alkaloids and increased the susceptibility of these plants against insects [110]. Salt stress reduced the photosynthesis and photosynthates contents in plants and this reduction might also be detrimental for the endophyte growth [72,73]. The soil salinity reduced the plant acquisition of nutrients such as nitrogen and phosphorus, and low levels of these nutrients in plants can alter the endophyte growth and production of alkaloids [29,111,112]. Salt stress associated with sodium produced water deficit (due to the excessive accumulation of sodium anions within plant cells) and reduced the uptake and transport of essential ions (e.g., potassium, calcium) [113]. There is a lack of evidence showing whether the salt-mediated water deficit and altered ion exchange directly affect endophytes. Further experiments might explore this possibility. 

## 4. Endophyte-Based Mechanisms of Plant Protection against Global Change Factors

*Epichloë* endophytes confer certain stress-protective mechanisms to plant hosts that may counteract the detrimental effects of the environmental factors associated with global change (Figure 2).

*Epichloë* endophytes can enhance the antioxidant contents in plants [19]. Antioxidants efficiently scavenge ROS and include several enzymatic and non-enzymatic compounds such as superoxide dismutase, catalase, peroxidases, glutathione, ascorbic acid, and proline [114,115]. In an experiment that included ozone as a treatment, the endophyte presence increased the content of proline antioxidants in plants, and this was associated with reduced levels of oxidate damage [116]. Similarly, under drought stress, endophytes reduced the oxidative stress in plants which was correlated with increased concentrations of several antioxidants [117]. The levels of the polyol mannitol, which can be produced by endophytes, were elevated in symbiotic plants that were subjected to drought stress [56,118]. The accumulation of mannitol (and also *Epichloë*-derived alkaloids) in drought situations may reduce the osmotic potential in plants and prevent the dehydration of cells [56]. Regarding flooding, endophytes increased the concentration of proline antioxidants in *H. brevisubulatum* plants, which was linked with low levels of oxidative stress [68]. Similar endophyte-mediated increases in proline levels were reported in certain genotypes of *F. arundinacea* plants subjected to the same stress [67]. In saline soils, the antioxidant capacity of *H. brevisubulatum* plants was enhanced by the endophyte presence [72].

*Epichloë* can reduce the concentration of defence-related hormones in plants. This reduction may prevent the induction of plant defence responses that inhibit the presence of endophytes within plant tissues [16]. As mentioned, plant defence responses associated with salicylic acid and jasmonic acid hormones are induced by global change stresses including ozone, temperature, and salinity (see for instance [90,95]). Experimental results have shown that *Epichloë* endophytes manipulate the concentrations of these phytohormones in the presence and absence of stresses. For instance, absent of any stress, endophytes reduced the concentration of salicylic acid in *L. multiflorum* plants [97,119]. Similarly, in the presence of stress, endophytes reduced the concentration of jasmonic acid and supressed part of the associated signalling pathway in *A. inebrians* plants [93]. Similar suppression of defence-related phytohormones by beneficial microorganisms have been documented in other symbiotic systems such as that between plants and mycorrhizal fungi [120,121]. The study of the interaction between *Epichloë* endophytes and stress-protective hormones has commenced. Drought stress increased the levels of the stress-protective hormone abscisic acid in endophyte-symbiotic *F. arundinacea* plants (although endophyte-free plants were not included in this study) [122]. Furthermore, an exogenous application of this hormone on *A. inebrians* plants increased the observed endophyte-mediated plant growth promotion in the presence of drought [123]. 

In the absence of stress, *Epichloë* endophytes induce multiple molecular changes in their hosts that may render plants sensitive or tolerant to global change stresses. In *L. perenne*, endophytes increased the expression of genes involved in cold/heat responses that changed the perception of plants to temperature stresses. In the latter study, the fungus also increased the expression of plant genes associated with the biosynthesis of raffinose oligosaccharides, which are temperature-protective metabolites [91]. The antioxidant contents in plants were also increased by endophytes in the absence of stress [124]. Furthermore, endophytes enhanced the levels of photosynthesis and upregulated several genes associated with this function in *A. inebrians* plants that were not exposed to stress [57]. In the presence of stress, *Epichloë* can induce certain responses that may help alleviate (perhaps quickly) the detrimental effects of global change stresses. In response to cold stress, endophytes increased the expression of genes coding for phytochrome and ethylene receptor proteins that are involved in the acclimatization of plants to low temperatures [125]. Under drought stress, endophyte presence stimulated the expression of plant genes coding for dehydrin and heat shock proteins that are known to prevent the cellular damage caused by stresses [126,127,128]. Furthermore, photosynthesis levels and the expression of several genes associated with the photosynthesis process were increased by the endophyte presence in *A. inebrians* plants in response to drought stress [57]. Similar outcomes in photosynthesis rates were reported in *H. brevisubulatum* plants that grew in soil with high salt contents [72]. In this species, endophytes also reduced the plant uptake of sodium ions and improved the plant endowment of nitrogen, phosphorus, and potassium in salt stress situations [72,112]. Similarly, the uptake of sodium (and chloride) ions by *F. arundinacea* and *Festuca pratensis* plants subjected to salt stress were also decreased by their associated *Epichloë* endophytes [129]. Furthermore, endophytes increased the diameter of xylem and phloem cells in plants that experienced salt stress. These anatomical changes were correlated with reduced levels of water loss in plants [113]. 

## 5. Concluding Remarks and Future Perspectives

We summarised evidence showing that environmental factors associated with global change influenced plant–*Epichloë* symbioses through compromising plant and endophyte traits and the symbiosis as well. Under the influence of global change factors, plant responses were mostly positively regulated by endophytes. However, negative effects of these factors were also documented. For example, combinations of high temperatures with humidity were associated with reductions in endophyte viability in seeds. In other cases, the incidence of environmental factors (e.g., ozone) impaired the benefits conferred by endophytes to plants. Although most of the research has been performed at individual level with few examples at population level (Figure 1), it is likely that global change factors exert substantial effects on the distribution and abundance of plant-endophyte symbioses in nature. This is particularly clear in situations where the factors turn beneficial symbioses into detrimental (i.e., parasitic) associations that eventually will be selected against. Additionally, there is an increasing interest in understanding whether vertically transmitted endophytes induce transgenerational effects on their plant hosts in the context of global change [130]. This has been only investigated in relation to ozone and drought, with no studies so far regarding other global change factors such as CO_2_, temperature, flooding, or salinity (Figure 1). We need further long-term manipulative experiments to determine, for instance, the effects of multiple and simultaneous global change factors on both plants and endophytes at individual level, and in the dynamics of endophyte-symbiotic plants. 

We posited that the induction of certain plant responses by global change factors would explain the effects of these factors on plant–*Epichloë* symbioses. These plant responses included the enhanced contents of ROS/defence-related hormones, and reduced levels of photosynthesis/nutrients (Figure 2). The direct effects of global change factors on *Epichloë* endophytes have been rarely studied. This may be because endophytes that are exclusively vertically transmitted do not present growth stages outside plants, thus the effects of environmental factors on the fungus cannot be easily separated from the effects on plants. However, the evaluation of endophyte transcriptomes and gene-edited endophytes are interesting approaches to improve the understanding of the direct effects the global change factors on the fungus [131,132]. We described the mechanisms by which endophytes may counteract the detrimental effects of the global change factors. These mechanisms included the endophyte ability to increase the plant antioxidant contents, reduce defence-related phytohormone concentrations, and increase the photosynthesis rates and plant uptake of nutrients (Figure 2). Further experiments will be necessary to evaluate if endophytes can increase the levels of stress-related phytohormones [133]. Enhanced levels of these hormones may increase the response symbiotic plants to stresses including those associated with the global change [134]. 

## Data Availability

Not applicable.
